# Predicting the distribution pattern changes of dye plant habitats caused by climate change

**DOI:** 10.3389/fpls.2024.1364481

**Published:** 2024-06-12

**Authors:** Jingpeng Duan, Jing Liu, Zhihuan Huang

**Affiliations:** ^1^College of Architecture and Design, University of South China, Hengyang, China; ^2^School of Life Sciences, Central China Normal University, Wuhan, China

**Keywords:** dye plants, climate change, MaxEnt, environmental factors, potential suitable habitats

## Abstract

Climate change has accelerated the habitat loss and fragmentation of wildlife. Dye plants of “Fengxiang dyeing” are important indigenous natural resources for traditional printing and dyeing craft in southwest China, is of practical and cultural importance for dozens of ethnic minorities. However, lack of the spatial distribution information of these plants has hampered holistic and efficient conservation management measures. We analyzed the potentially suitable areas of four dye plants (*Liquidambar formosana*, *Strobilanthes cusia*, *Persicaria tinctoria* and *Indigofera tinctoria*) necessary for “Fengxiang dyeing” based on their geographical distribution sites under different climatic situations using the maximum entropy (MaxEnt) model. The results showed that temperature, precipitation and elevation were the most important factors affecting the suitable geographical areas of the four dye plants. Under the current climate conditions, the overlapping suitable habitat areas of the four plants were mainly in the four southern provinces of China, including Guizhou, Guangxi, Guangdong and Hainan. *L. formosana* was used as the base plant for combination with the other three plants under the two future climate scenarios (SSP126 and SSP585), and the overlapping suitable habitat areas of the obtained seven combination patterns were considered suitable for potential craft development. Five patterns showed an increase, while two patterns showed a decreasing trend with the increasing carbon emission. The prediction results showed that the overlapping suitable habitat center of the four plants will gradually move to the northeast, indicating that the overlapping suitable habitat area and craft distribution area will be changed. These results provide the basis for understanding the spatial distribution pattern changes of dye plants caused by climate change and establishing measures for protecting and developing printing and dyeing craft.

## 1 Introduction

The ecological environment profoundly affects the formation of ethnic communities (Yang, 2007; Siegert et al., 2023). The ethnic minority residents such as Bouyei, Yao and Miao, have lived in mountainous and hilly areas within the monsoon-influenced humid subtropical climate zone in southern Guizhou, China, for generations. Due to inconvenient transportation and isolated environments, the ethnic tribes are relatively independent, necessitating self-sufficiency in producing material resources. This independent living environment has led to many unique indigenous cultural practices, among which “Fengxiang Dyeing” is the most attractive intangible cultural heritage (Liu et al., 2021). “Fengxiang Dyeing” is a traditional dyeing craft for extracting Liquidambaris Resina from *Liquidambar formosana* Hance to serve as a resist agent for combination with blue dye obtained from plants (such as *Strobilanthes cusia* Kuntze, *Persicaria tinctoria* Spach and *Indigofera tinctoria* L.) (Qin, 2014; Zhou, 2015; Li, 2017). Relying on the natural dyes and resist agent, indigenous dyemakers are able to produce artistic and utilitarian ornamental design for dress and furnish (Liu et al., 2021). The “Fengxiang Dyeing” history can be traced back to the Song Dynasty, with records found in the ancient Bouyei song “Mo Shan Zhuang·Wen Zhuang” and the “History of Song”(in volume 493): “Nanning Prefecture (now Huishui County of Guizhou)”, known for its production of horses, cinnabar, and Liquidambaris Resina dyed cloth (Qin, 2014). The “Fengxiang Dyeing” craft is often described as blue-and-white porcelain on canvas for its exquisite and elegant designs. The State Council of China officially listed “Fengxiang Dyeing” in the second batch of national intangible cultural heritage in 2008 (Liu et al., 2021).

Climate influences species distributions by imposing geographic limits based on species’ physiological tolerances to temperature and precipitation gradients (i.e., the fundamental niche) (Stevens, 1989; Gaston and Spicer, 2001). Along with climate change, the geographic limits of tolerable climatic conditions shift in space, forcing species to move or face niche mismatch, resulting a decline in population, alter geographical distribution patterns and even extinction (Bellard et al., 2012; Pecl et al., 2017; Liang et al., 2018). Many researches have shown that climate change has accelerated the habitat loss and fragmentation of plants and animals. For example, Hamilton et al. (2024) found the distribution of five berry plant species in southwestern Alaska is expected to shrink under almost all future climate scenarios tested. Separate research revealed that climate change negatively impacts the distribution of Gobi Bear (*Ursus arctos gobiensis*) by shrink the distribution of the main plant food resources in its habitat (Qin et al., 2020). At present, research of climate change impact on plant distribution mainly focuses on ornamental, edible and medicinal, and rare and endangered species, little is known about potential suitable habitat changes of indigenous culture-related plant species response to climate change (Siegert et al., 2023). Therefore, evaluating the effect of climate change on the spatial distribution of dye plant species will help optimize the use of these resources and develop effective management measures to protect “Fengxiang Dyeing”.

Species Distribution Models (SDMs) have been used to predict the geographical distribution of species over different periods (Ferrier and Guisan, 2006; Aguilar-Soto et al., 2015). The currently popular SDMs include MaxEnt, GARP, Bioclim, Domain and ENFA (Peng and Feng, 2022). MaxEnt (Maximum Entropy) is a general machine-learning method that utilizes environmental climate factors for quantitative analysis. Compared to other models, MaxEnt requires only species occurrence data and performs well with small sample sizes (Wisz et al., 2008; Rhoden et al., 2017). MaxEnt also has the advantage of allowing the use of continuous and categorical variables (Baldwin, 2009). As an efficient tool for species distribution model analysis, MaxEnt has been widely used in climate change, species conservation and biological invasion studies (Moreno-Amat et al., 2015; Sahragard and Chahouki, 2015; Sharma et al., 2018; Li et al., 2022).

The main distribution areas of several dye plants are expected to change with the increasing global temperature. We speculate that the temperature increase may diminish the suitable growth areas of several dye plants, and the distribution center may shift to the north. The development of “Fengxiang Dyeing” highly depends on the nearby acquisition of dye plant resources (Ai, 2020). Climate change may lead to a shift of suitable habitat area of dye plants away the craft center, which directly affected the acquisition cost of basic dye materials. Therefore, it is important to study the temporal and spatial distribution patterns of dye plants caused by climate change for the development and protection of traditional dyeing crafts.

To predict the suitable distribution areas of “Fengxiang Dyeing” dye plants under climate change, we: (1) predicted the distribution of suitable areas of the four dye plants (*L. formosana*; *S. cusia*, *P. tinctoria*, and *I. tinctoria*) under the current climate conditions using the MaxEnt model, and calculated their overlapping suitable areas as the “Fengxiang Dyeing” craft suitable areas; (2) predicted the distribution of the four dye plants under future climate scenarios and the trend of their overlapping suitable areas. We used *L. formosana* as the base dye plant for combination with at least one to at most three blue dye plants (*S. cusia*, *P. tinctoria*, and *I. tinctoria*), and obtained seven combinations of the overlapping suitable zones as the craft suitable areas. We then calculated the trend of their overlapping suitable areas under the future climate scenarios. The findings provide the basis for understanding the spatial distribution pattern changes of dyeing plants caused by climate change and establishing measures for protecting and developing printing and dying craft.

## 2 Materials and methods

### 2.1 Occurrence records

**Figure 1:**
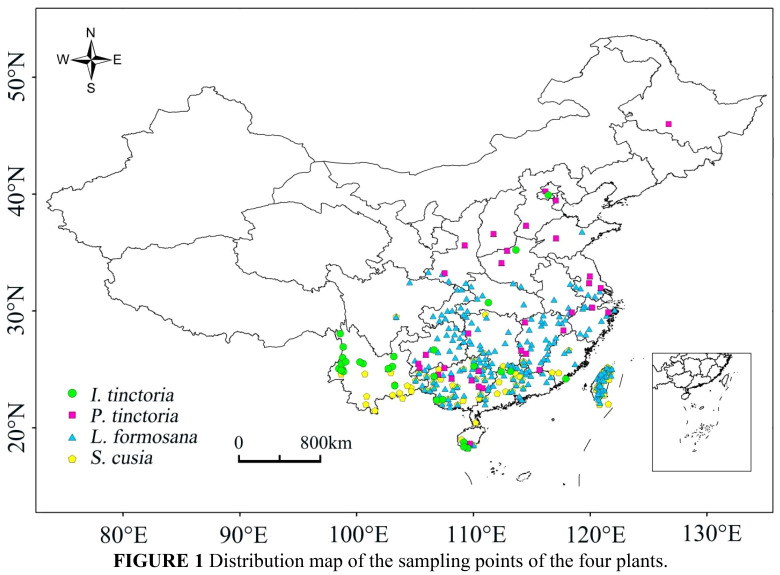
Distribution map of the sampling points of the four plants.

The geographical distribution point data of the four plants were sourced from the Global Biodiversity Information Facility (http://www.gbif.org), with reference to the National Specimen Information Infrastructure (http://www.nsii.org.cn) and the Chinese Virtual Herbarium (https://www.cvh.ac.cn). Spatial filtering of the species distribution data was conducted to avoid overfitting caused by spatial sampling bias and to improve the precision of predictions (Phillips et al., 2009; Veloz, 2009; Hijmans, 2012; Boria et al., 2014). The spatial thinning tool SDMtoolbox_v2.5 was used to reduce the high spatial autocorrelation in the distribution of the four plants (Brown, 2014; Brown et al., 2017), with a resolution of 15 km (**Figure 1**). After data filtering, the data points obtained for the four plants were as follows: *L. formosana*: 242; *P. tinctoria*: 33; *S. cusia*: 89; *I. tinctoria*: 29. These data were saved in coma-separated values (CSV) format.

### 2.2 Environmental variables

Nineteen bioclimatic variables (current from 1970–2000) and elevation data were obtained from WorldClim 2.1(https://www.worldclim.com). Digital Elevation Model (DEM) data were sourced from (http://www.gscloud.cn), and slope and aspect data were extracted using ArcGIS 10.8 (https://support.esri.com). Seven soil quality data sets were acquired from the Food and Agriculture Organization of the United Nations (https://www.fao.org/home/en). Land cover type data were obtained from the International Steering Committee for Global Mapping (ISCGM) (https://globalmaps.github.io/glcnmo.html). The 30 environmental variable datasets were rescaled to a resolution of 2.5 arc-minutes using ArcGIS, and then converted to ASCII format after coordinate system standardization. The study area was limited to China, with layers clipped to the administrative boundaries of China using map data from the National Geomatics Center of China (http://www.ngcc.cn/ngcc).

Future climate data were selected from the BBC-CSM2-MR model under the CMIP6 scenario, which is more suitable for predicting climatic distribution due to its accuracy in simulating global temperature and precipitation distribution, atmospheric radiation and ocean budget. Therefore, the BCC-CSM2-MR model’s prediction of precipitation and temperature performance in China is particularly reliable (Wu et al., 2019). The specific scenarios were: (1) SSP126, representing a low development and low emission scenario (Liu et al., 2022a), and (2) SSP585, representing a high development and high emission scenario (Ma et al., 2022a). The two periods represented long-term average climate conditions for 2041–2060 (referred to as 2050) and 2061–2080 (referred to as 2070). To minimize collinearity among all environmental factors, we sampled environmental factor data using ArcGIS 10.8. Highly correlated environmental factor variables (r^2^ ≥ |0.8|) were removed using the Pearson analysis feature of IBM SPSS Statistics 26 (Wei et al., 2020; Gao et al., 2022) based on the contribution of environmental factors in the MaxEnt model, retaining important variables with ecological significance (Kaky and Gilbert, 2016; He et al., 2020) (**Table 1**).

**Table 1:** The selected environmental factors.

Abbreviation	Mean	Note
BIO1	Annual Mean Temperature	1/4
BIO2	Mean Diurnal Range (Mean of monthly (max temp - min temp))	1/2/4
BIO3	lsothermality (BIO2/BIO7) (x100)	1/2/3
BIO4	Temperature Seasonality (standard deviation x 100)	4
BIO5	Max Temperature of Warmest Month	3
BIO6	Min Temperature of Coldest	1/2
BIO7	Temperature Annual Range (BIO5-BIO6)	1
BIO8	Mean Temperature of Wettest Quarter	2
BIO10	Mean Temperature of Warmest Quarter	3
BIO11	Mean Temperature of Coldest Quarter	1
BIO12	Annual Precipitation	1/3
BIO13	Precipitation of Wettest Month	2
BIO14	Precipitation of Driest Month	2/4
BIO15	Precipitation Seasonality (Coefficient of Variation)	2/3/4
BIO16	Precipitation of Wettest Quarter	4
BIO17	Precipitation of Driest Quarter	1
BIO18	Precipitation of Warmest Quarter	3/4
Sq1	Nutrient availability	3/4
Sq2	Nutrient retention capacity	1/2/3
Sq3	Rooting conditions	1/2/3/4
Sq4	Oxygen availability to roots	1/2/3/4
Sq5	Excess salts	2/3/4
Sq6	Toxicity	3/4
Sq7	Workability (constraining field management)	3/4
Elevation	Elevation	1/2/3/4
Slop	Slop	1/2/3/4
Aspect	Aspect	1/2/3/4
Land Cover	Types of land suitable for plant growth	1/2/3/4

1, 2, 3 and 4 indicate that the factor is included in the *Strobilanthes cusia*, *Liquidambar formosana*, *Persicaria tinctoria*, and *Indigofera tinctoria* models, respectively.

### 2.3 Model simulation and evaluation

The distribution point data (in CSV format) of each plant and environmental factor (in ASCII format) were imported into MaxEnt 3.4.4 (http://biodiversityinformatics.amnh.org) (Phillips et al., 2009), and 25% of all data points were selected for model validation and 75% for model training. Ten-fold cross-validation was used with the repetition type set to Bootstrap, the regularization multiplier set to the default of 1, and the background points and maximum iterations set to 10,000 and 500, respectively. A training presence threshold of 10% and the Cloglog output format were used, and the model analysis was repeated 10 times to obtain an average value.

The accuracy of the model results was referenced to the Area Under the Receiver Operating Characteristic Curve (AUC). The higher the AUC value (closer to 1), the higher the accuracy of the model (Fielding and Bell, 1997; Phillips et al., 2006). Additionally, a Jackknife test was employed to analyze the contribution rate of model factors (Ma et al., 2022b).

### 2.4 Spatial pattern changes of the overlapping area

ArcGIS was used to create two types of species suitability distribution maps: (1) An overlapping probability map obtained by superimposing the MaxEnt prediction results of the four plants to discuss the spatial pattern changes of the distribution of several plants under current and future climate scenarios (El-Gabbas et al., 2016). (2) A “binary map” created by reclassifying the prediction results of each plant, with a probability value of 30% as the threshold, whereby areas above 30% were considered suitable habitats (denoted by “1”), and areas below 30% were considered non-suitable habitats (denoted by “0”). After reclassification, the “binary map” layers were superimposed to create a species richness map, with “0” indicating no species distribution and higher numbers indicating more overlapping species (Qiu et al., 2020).

Different combination patterns of the “binary map” of *L. formosana* with those of the other three plants were superimposed to obtain different overlapping suitable area patterns, which served as a basis for assessing the potential suitable area changes for craft development. SDMtoolbox_v2.5 was used to calculate the centroid migration of suitable areas under different climate scenarios.

## 3 Results

### 3.1 Model performance and variables’ contribution

The average AUC values for all four plants were above 0.9 under current and future climate scenarios, suggesting good model performance (**Table 2**). This is because AUC values greater than 0.9 indicate a high precision of the predictive results (Swets, 1988).

**Table 2:** Area under the receiver operating characteristic (ROC) curve under different climate scenarios.

Species	Years	Climate scenario	Training AUC(Mean ± SE)
*L. formosana*	current	–	0.946 ± 0.004
	2050	SSP126	0.945 ± 0.004
	2070	SSP126	0.944 ± 0.003
	2050	SSP585	0.946 ± 0.004
	2070	SSP585	0.946 ± 0.002
*S. cusia*	current	–	0.977 ± 0.002
	2050	SSP126	0.981 ± 0.003
	2070	SSP126	0.980 ± 0.004
	2050	SSP585	0.979 ± 0.003
	2070	SSP585	0.977 ± 0.003
*P. tinctoria*	current	–	0.961 ± 0.011
	2050	SSP126	0.974 ± 0.008
	2070	SSP126	0.965 ± 0.005
	2050	SSP585	0.970 ± 0.012
	2070	SSP585	0.960 ± 0.007
*I. tinctoria*	current	–	0.987 ± 0.005
	2050	SSP126	0.987 ± 0.005
	2070	SSP126	0.986 ± 0.008
	2050	SSP585	0.986 ± 0.007
	2070	SSP585	0.988 ± 0.004

Referring to the Jackknife gain values of the environmental factors tested for each plant (**Supplementary Figure 1**), and considering the percent contribution and permutation importance of the four plant environmental factors (**Supplementary Tables 1–4**), we chose the top four important environmental factors affecting each plant individually, for a total of sixteen (**Table 3**). The primary environmental factors affecting *L. formosana* included the lowest temperature of the coldest month, the monthly mean of the diurnal temperature range, the precipitation of the driest month, and the precipitation of the wettest month, with a cumulative contribution rate of 82.4%; For *S. cusia*, the main factors were the annual temperature range, the average temperature of the coldest quarter, annual precipitation, and mean annual temperature, with a cumulative contribution rate of 63.1%; For *P. tinctoria*, the factors were the lowest temperature of the coldest month, precipitation of the hottest quarter, annual precipitation and altitude, with a cumulative contribution rate of 54.6%; The factors for *I. tinctoria* included seasonality of temperature (standard deviation × 100), mean annual temperature, precipitation of the driest month, and precipitation of the hottest quarter, with a cumulative contribution rate of 62.1%.

**Table 3:** Main environmental factors and their contribution rates for the four plants.

Species	Code	Variable description	Unit	Regularized training gain	Percent contribution
*L. formosana*	BIO_6	Min Temperature of Coldest Month	°C	1.39	8.6
	BIO_2	Mean Diurnal Range (Mean of monthly (max temp - min temp))	°C	1.37	3.2
	BIO_14	Precipitation of Driest Month	mm	1.34	69.2
	BIO_13	Precipitation of Wettest Month	mm	1.24	1.4
*S. cusia*	BIO_7	Temperature Annual Range (BIO5-BIO6)	°C	2.2	31.7
	BIO_11	Mean Temperature of Coldest Quarter	°C	2.1	5.8
	BIO_12	Annual Precipitation	mm	1.9	22.6
	BIO_1	Annual Mean Temperature	°C	1.8	3
*P. tinctoria*	BIO_6	Min Temperature of Coldest Month	°C	0.85	16.0
	BIO_18	Precipitation of Warmest Quarter	mm	0.75	12.0
	BIO_12	Annual Precipitation	mm	0.74	16.1
	e	Elevation	m	0.65	10.5
*I. tinctoria*	BIO_4	Temperature Seasonality (standard deviation ×100)	–	1.4	33.2
	BIO_1	Annual Mean Temperature	°C	1.2	8.1
	BIO_14	Precipitation of Driest Month	mm	1.0	15.6
	BIO_18	Precipitation of Warmest Quarter	mm	0.8	5.2

### 3.2 The overlapping areas under current climate scenarios

**Figure 2:**
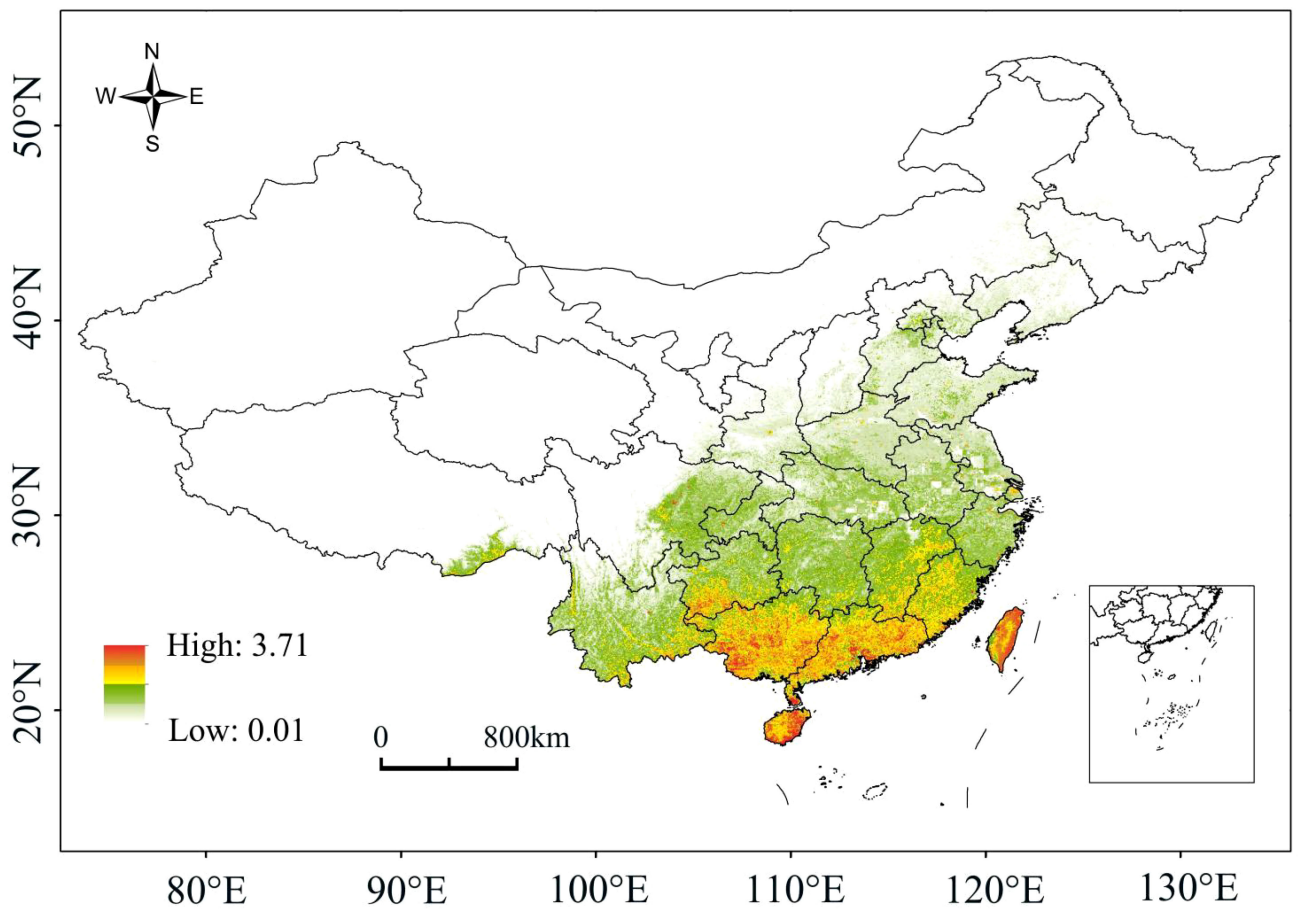
A probability map of the overlapping suitable areas.

**Figure 3:**
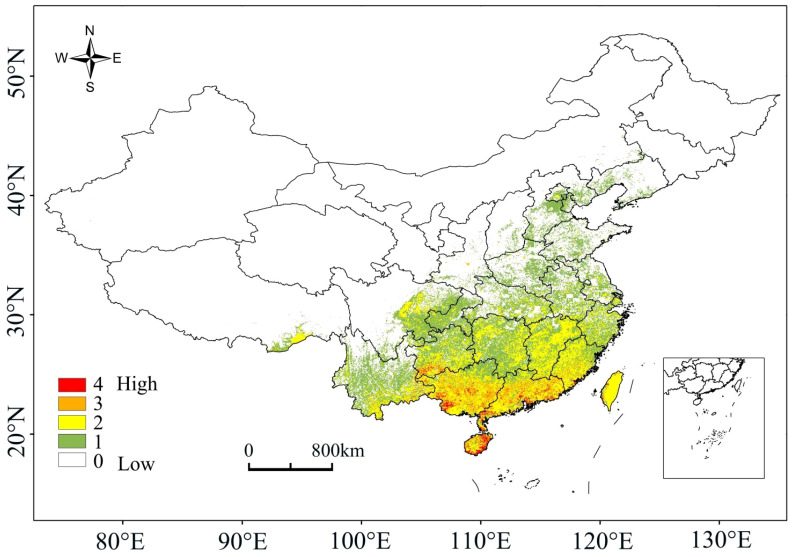
A species richness map.

Under the current climate conditions, the high-probability overlapping suitable habitats of the four plants were mainly located in Hainan, Guangdong, Guangxi, Taiwan and southern Guizhou, with scattered distributions also present in Jiangxi, Fujian, Hunan and Yunnan (**Figure 2**). For species richness, Hainan, Guangdong, Guangxi and southern Guizhou had the most areas with overlapping distributions of the four plants, with a few parts in Jiangxi, Fujian, Hunan and Yunnan (**Figure 3**). The high-probability overlapping suitable habitats corresponded well with areas of high species richness, predominantly situated in lower latitude regions. Although Taiwan had high probability values, most regions only had two species present.

### 3.3 Dynamic changes of potential habitats under future climate scenarios

#### 3.3.1 Changes in the distribution patterns of potential habitats

The changes in suitable habitat areas of the four plants exhibited different patterns under the two future climate scenarios (SSP126 and SSP585) for the years 2050 and 2070. *L. formosana* showed an increasing trend in suitable habitat areas under the SSP126 scenario and a decreasing trend under the SSP585 scenario, while *S. cusia* showed a decreasing trend under the SSP126 scenario and an increasing trend under the SSP585 scenario. Conversely, *P. tinctoria* exhibited a decreasing trend under both future scenarios, while *I. tinctoria* showed an increasing trend under both future scenarios (**Table 4**).

**Table 4:** Suitable habitat area changes of the four plants under different climate scenarios.

Years	Climate scenario	Area(×10^4^km^2^)			
		*L. formosana*	*S. cusia*	*P. tinctoria*	*I. tinctoria*
Current	–	134.52	38.94	89.16	30.56
2050	SSP126	140.54	38.31	53.69	31.50
2070	SSP126	146.22	37.53	87.77	31.16
2050	SSP585	130.09	41.74	66.38	36.68
2070	SSP585	134.51	43.65	80.99	31.40

The essential raw material for “Fengxiang Dyeing” is the Liquidambaris Resina from *L. formosana*. *L. formosana* with any one or more blue dye plants can fulfill the dye resource requirements for “Fengxiang Dyeing”. Therefore, we obtained combinations using *L. formosana* as a base plant, with the other three plants. Predicting the changes in suitable areas of these combinations under the future climate scenarios—whether increasing or decreasing, can help skill inheritors better access dye plant resources and cope with changes in distribution patterns caused by climate change. Seven combinations were obtained, including *L. formosana* + *S. cusia* (1), *L. formosana* + *P. tinctoria* (2), *L. formosana* + *I. tinctoria* (3), *L. formosana* + *S. cusia* + *P. tinctoria* (4), *L. formosana* + *S. cusia* + *I. tinctoria* (5), *L. formosana* + *P. tinctoria* + *I. tinctoria* (6), and *L. formosana* + *S. cusia* + *P. tinctoria* + *I. tinctoria* (7) (**Table 5**). The overlapping suitable habitats of these seven combinations were considered potential distribution areas for craft development. Under the SSP126 climate scenario in 2050, combinations 1, 6 and 7 showed a slight decreasing trend in suitable habitat area, while combinations 2 and 4 showed a significant decrease, and combinations 3 and 5 exhibited a slight increase. Under the SSP126 climate scenario in 2070, combinations 1, 4 and 5 showed a slight decreasing trend, while combinations 2, 3, 6 and 7 showed an increasing trend, and combination 2 showed a significant change. Under the SSP585 climate scenario, in 2050 and 2070, combinations 1, 3, 5, 6 and 7 exhibited an increasing trend, which was more evident in combinations 3 and 5, while combinations 2 and 4 showed a decreasing trend.

**Table 5:** Suitable habitat area changes of the seven combinations under different climate scenarios.

Years	Climate scenario	Area(×10^4^ km^2)^						
		1	2	3	4	5	6	7
Current	–	33.71	51.53	10.07	13.52	6.10	5.77	3.36
2050	SSP126	32.79	32.27	12.43	8.24	7.47	4.84	2.99
2070	SSP126	32.39	54.25	10.23	12.45	5.90	6.04	3.78
2050	SSP585	35.13	41.30	14.75	11.65	9.38	7.09	4.38
2070	SSP585	35.83	46.90	10.27	13.26	6.90	5.86	4.02

1–7 represent the seven combinations which are; 1. *L. formosana* + *S. cusia*; 2. *L. formosana* + *P. tinctoria*; 3. *L. formosana* + *I. tinctoria*; 4. *L. formosana* + *S. cusia* + *P. tinctoria*; 5. *L. formosana* + *S. cusia* + *I. tinctoria*; 6. *L. formosana* + *P. tinctoria* + *I. tinctoria*; 7. *L. formosana* + *S. cusia* + *P. tinctoria* + *I. tinctoria*.

#### 3.3.2 Centroid migration of the overlapping areas under different climate scenarios

**Figure 4:**
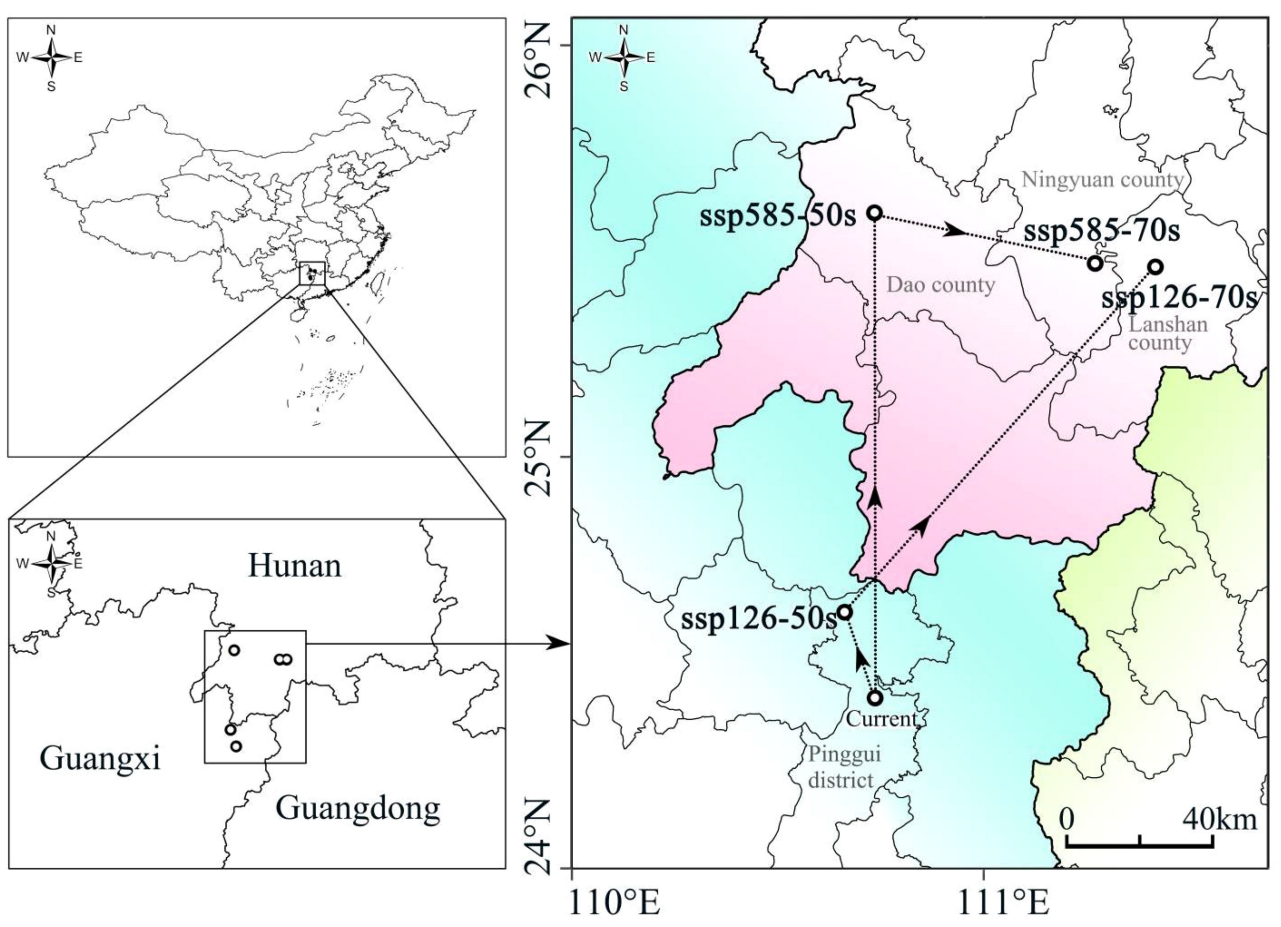
Centroid position of the superimposed suitable habitat areas of the four species under different climate scenarios.

The probability map of overlapping suitable habitats under current and future climate scenarios serves as the basis for studying dynamic changes. Under the current climate conditions, the centroid of the overlapping suitable habitats of the four plants was located in the central part of Pinggui District, Hezhou City (24°22’N, 111°30’E). The prediction showed that under the SSP126 scenario, the centroid will have moved to the northwestern part of Pinggui District, Hezhou City (24°34’N, 111°26’E) by 2050, and to the northwestern part of Lanshan County, Yongzhou City (25°26’N, 112°8’E) by 2070. Under the SSP585 scenario, the centroid will have moved to the western part of Dao County, Yongzhou City (25°33’N, 111°29’E) by 2050 and to the southeastern part of Ningyuan County, Yongzhou City (25°26’N, 112°2’E) by 2070. Overall, the centroid of suitable habitats moves in a northeast direction under both future climate scenarios (**Figure 4**).

## 4 Discussion

### 4.1 Distribution of current and future potential habitats

**Figure 5:**
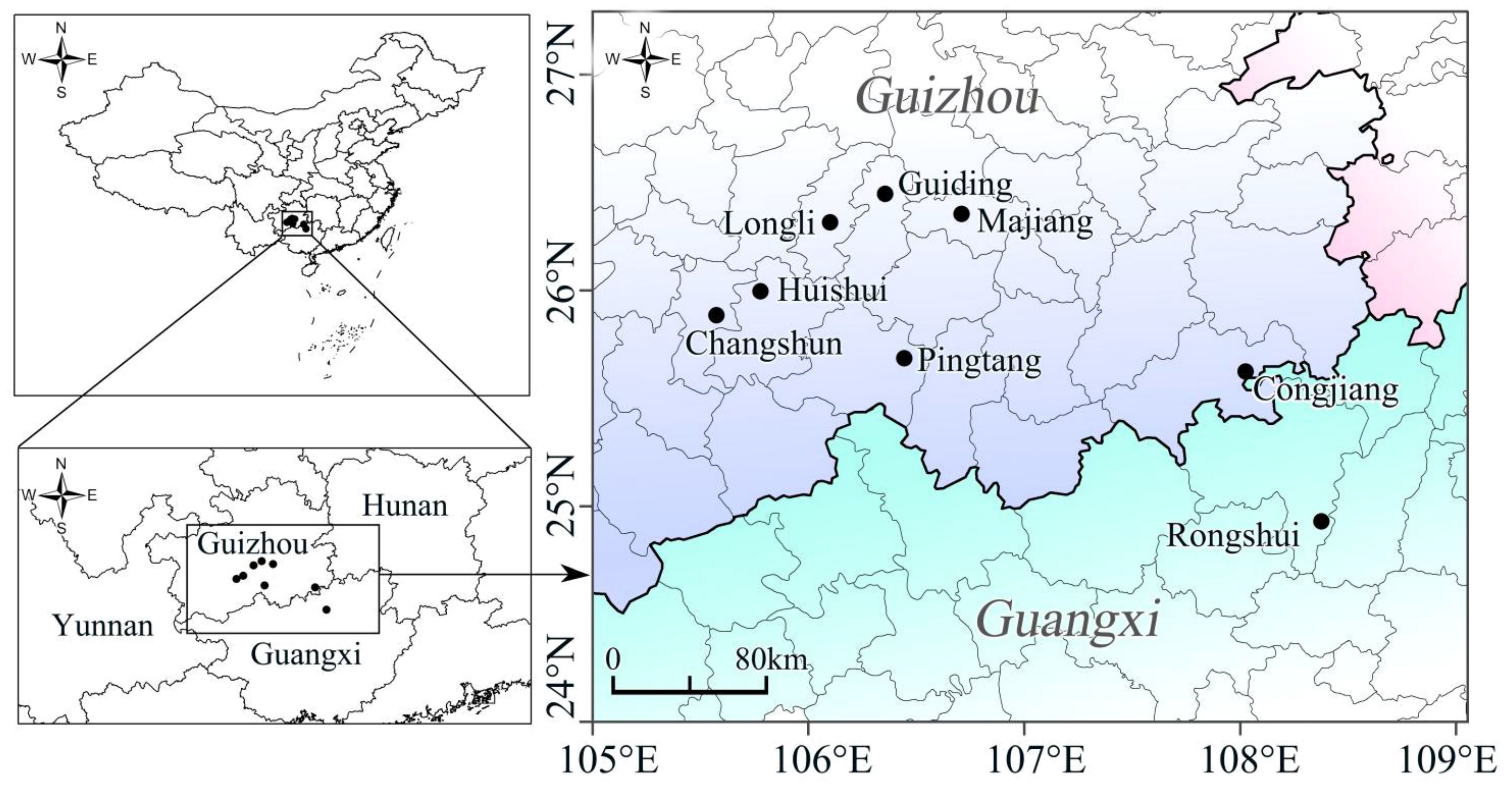
Distribution locations of the “Fengxiang Dyeing” craft.

Currently, the “Fengxiang Dyeing” craft is primarily distributed across five counties in the Buyei and Miao Autonomous Prefecture, the south of Guizhou (Huishui, Pingtang, Guiding, Longli, and Changshun); Also in the Majiang and Congjiang counties, the southeast of Guizhou, Miao and Dong Autonomous Prefecture in China (**Figure 5**). Among these, the craft is best preserved in Yashui Town of Huishui County and Heba Village of Majiang County and relatively intact in Rongshui Miao Autonomous County of Guangxi (Qin, 2014; Jia and Jia, 2018; Zhang and Nie, 2019). The high-probability areas on the probability map of the overlapping suitable habitats of the four plants were located in southern Guizhou, Hainan, Guangdong, Guangxi, and Taiwan. A comparison of the current distribution of the craft with the probability map of the current climate scenarios for the four plants revealed a high degree of overlap in southern Guizhou. The areas with the highest species richness were primarily in southern Guizhou, Hainan, Guangdong and Guangxi, which are also densely populated regions of ethnic minorities in southern China (Dong, 2012).

The predicted changes in the distribution patterns of suitable habitats of each plant and the seven patterns of the overlapping suitable habitats indicated varying trends of increase or decrease for different plants and combinations. Moreover, the perditions showed that the centroid of the suitable habitats will move towards higher altitudes as the climate warms.

According to our results, *L. formosana* can be combined with three blue dye plants to meet the needs of the “Fengxiang Dyeing” craft, and the seven derived patterns of the overlapping suitable habitats can all be considered potential areas for craft development. Between 2050 and 2070, the overlapping suitable habitat changes in the areas of the seven patterns showed different trends under two future climate scenarios with increasing carbon emissions, with five patterns showing an increasing trend and the other two showing a decrease. The centroid of the overlapping suitable areas of the four plants migrated to the northeast. Therefore, the range of the overlapping suitable areas of the four plants will also change with climate change, resulting changes of the coincidence position with the existing craft. Moreover, we found that under the two different future climate scenarios, the expansion of the suitable habitat areas will favor the supply of dye plants, but the accompanying reduction will also impact the craft regions. The northeastward migration of the suitable habitat centroid is favorable since the overlapping suitable area within the range of “Fengxiang Dyeing” craft distribution locations will have better overlap compared to the current climate scenarios. The location of the “Fengxiang Dyeing” craft near the distribution of dye plants is more conducive to the inheritance and conservation, while the deviation of dye plants increases the cost of acquisition. As a result, the changes in the range of the suitable habitats of the plants directly affect the supply and material security of the “Fengxiang Dyeing” dye plants. In the context of future climate change, it is considered to designate economic crop planting areas for dye plants to ensure the supply of dye plants. The appropriate locations can be selected based on the distribution patterns of future suitable growing areas for different combination patterns in the predicted results.

### 4.2 Environmental variables affecting the four dye plants' distribution

In general, the climatic conditions suitable for the survival of the four plants correspond to their common suitable climates in the subtropical climate zone of southern China and the fringe tropical climate zone. This region is characterized by high annual precipitation (1200–2000 mm), insignificant seasonal changes, and an average temperature of > 4°C in the coldest month (Gao et al., 2022; Qin et al., 2022). A certain temperature range is conducive to the growth of *L. formosana*, with the most contributive environmental factor being the precipitation of the driest month (above 25mm). The most critical environmental factor for *S. cusia* is the annual temperature range between 7–25°C at lower altitudes, while the highest contributing environmental factor for *P. tinctoria* is the lowest temperature of the coldest month (> -7°C). Areas with smaller seasonal temperature differences, with temperatures between 10–30°C, are suitable for the growth of *I. tinctoria*. Overall, temperature-related bioclimatic variables significantly impact the range of suitable habitats for the four dye plants (**Supplementary Figure 2**). As global temperatures rise in the future, suitable habitats for dye plants will migrate towards higher latitudes. Our climate scenarios predicted that the range of suitable habitats for the four dye plants might shift toward higher latitudes, consistent with the temperature-rising trend of the coldest month in higher latitudes (Miao et al., 2022). Besides temperature, precipitation is also an important factor affecting the geographical distribution of plant species, but it is more difficult to predict than temperature (Kendon et al., 2014; Salzmann, 2016). Research has shown that, in some regions, climate change can lead to increased precipitation with increasing latitude (Poujol et al., 2021), thereby affecting the adaptive pattern of plants. Recent survey reports on the geographical distribution of some wild animals and plants in the study area have also shown that many hygrophilic plant species are emerging in previously arid habitats (Liu et al., 2022b).

### 4.3 Importance of interspecific relationship in predicting species suitable areas

Predicting the potential habitat of species based on occurrence data and different environmental variables can provide important information for species conservation planning, degraded habitat reclamation projects, and evaluating the impact of human activities and climate change on the survival status of species (Thorn et al., 2009; Wilson et al., 2011; Qin et al., 2017). For example, predicting potential habitats for rare and endangered species can help identify environmental factors affecting their growth, discover potential future habitats, and develop reasonable conservation strategies (Gao et al., 2022; Lee et al., 2023). However, direct prediction cannot be conducted for research objects whose geographic distribution data cannot be obtained. Sometimes, changing perspectives is also an effective means. This study focused on four dye plants necessary for the “Fengxiang dyeing” craft. Our future climate scenarios predicted that the overlap between the suitable areas of the four dye plants and the craft will be changed. This is consistent with the mode of Qin et al. (2020), which showed that the Gobi Bear (*Ursus arctos gobiensis*) could not survive without the three main plant food resources in its habitat. Similarly, Gao et al. (2022) argued that the protection of endangered and rare plants should not only focus on the impact of climate change, habitat conditions, and their survival conditions but should also consider the potential distribution of other species that have important interactions with them (such as those involved in cooperation, competition and predation interactions). The dye plants required for the “Fengxiang dyeing” craft are not singular; therefore, it is more appropriate to calculate their overlapping suitable growth areas. Therefore, our research provides a paradigm for predicting the suitable areas of species whose data are inconvenient to measure and recording their occurrence data (which can provide information on their essential survival resources and other ethnic cultures that rely on fixed material resources).

### 4.4 Limitations and uncertainties

The survival of dye plants is influenced by various factors, including physiological and ecological factors of species and other complex environmental factors such as extreme climate change, pests and diseases. Therefore, the prediction model could not include all of the factors. In addition, the species occurrence data used for the prediction was small and might have introduced some errors and uncertainties in the simulation results due to overfitting. Despite these shortcomings, we utilized MaxEnt to overlap the suitable areas of the four dye plants and obtained multiple combinations that served as suitable areas for the “Fengxiang dyeing” craft. This study comprehensively analyzed the appropriate habitats of the four dye plants and provided a vital link to developing policies and plans for the sustainable use and management of this important plant resource.

## 5 Conclusion

The “Fengxiang Dyeing” artifacts depend on dyes obtained from plants such as *P. tinctoria*, *S. cusia*, and *I. tinctoria*, cultivated and harvested locally (Ai, 2020). Unlike chemical dyes that decompose into various carcinogenic or allergenic substances, plant dyes are more environmentally friendly and healthy, making their promotion important today (Ren and Wang, 2011). Dye plant resources are one of the crucial factors for the development of traditional dyeing crafts; however, their range of suitable habitats is affected by climate change, potentially leading to inconsistencies with their current craft distribution locations. The prediction results showed that under various future climate scenarios, the spatio-temporal distribution patterns of the four dye plants would change according to environmental factors. The increase in temperature and changes in precipitation are causing the expansion of suitable habitats for dye plants toward higher latitudes and altitudes while causing a contraction trend in lower latitudes and altitudes. The development of the “Fengxiang Dyeing” craft mainly relies on the four dye plants, and future changes in the distribution patterns of the suitable habitats of these plants will affect the supply of raw materials. Plant habitats have been deteriorating due to the effects of climate change, limiting the protection and utilization of ethnobotanical resources. Thus, the results of this study provide a reference for delineating the protection scope of dye plants and developing economic planting zones for dye plants. The study also provides a new perspective on the protection of traditional culture.

## Data availability statement

The raw data supporting the conclusions of this article will be made available by the authors, without undue reservation.

## Author contributions

JD: Conceptualization, Data curation, Formal Analysis, Investigation, Methodology, Validation, Writing – original draft. JL: Conceptualization, Data curation, Formal Analysis, Investigation, Methodology, Validation, Writing – original draft. ZH: Conceptualization, Data curation, Formal Analysis, Funding acquisition, Investigation, Methodology, Validation, Writing – original draft, Writing – review & editing.
