# Supplementary material for: Predicting the distribution pattern changes of dye plant habitats caused by climate change
Source: Front Plant Sci. 2024 Jun 12;15:1364481. doi: 10.3389/fpls.2024.1364481 (PMC11210319; doi:10.3389/fpls.2024.1364481)
Supplement: Supplementary file 1 [file DataSheet_1.doc]

Supplementary Material

# Supplementary Figures and Tables

## Supplementary Figures


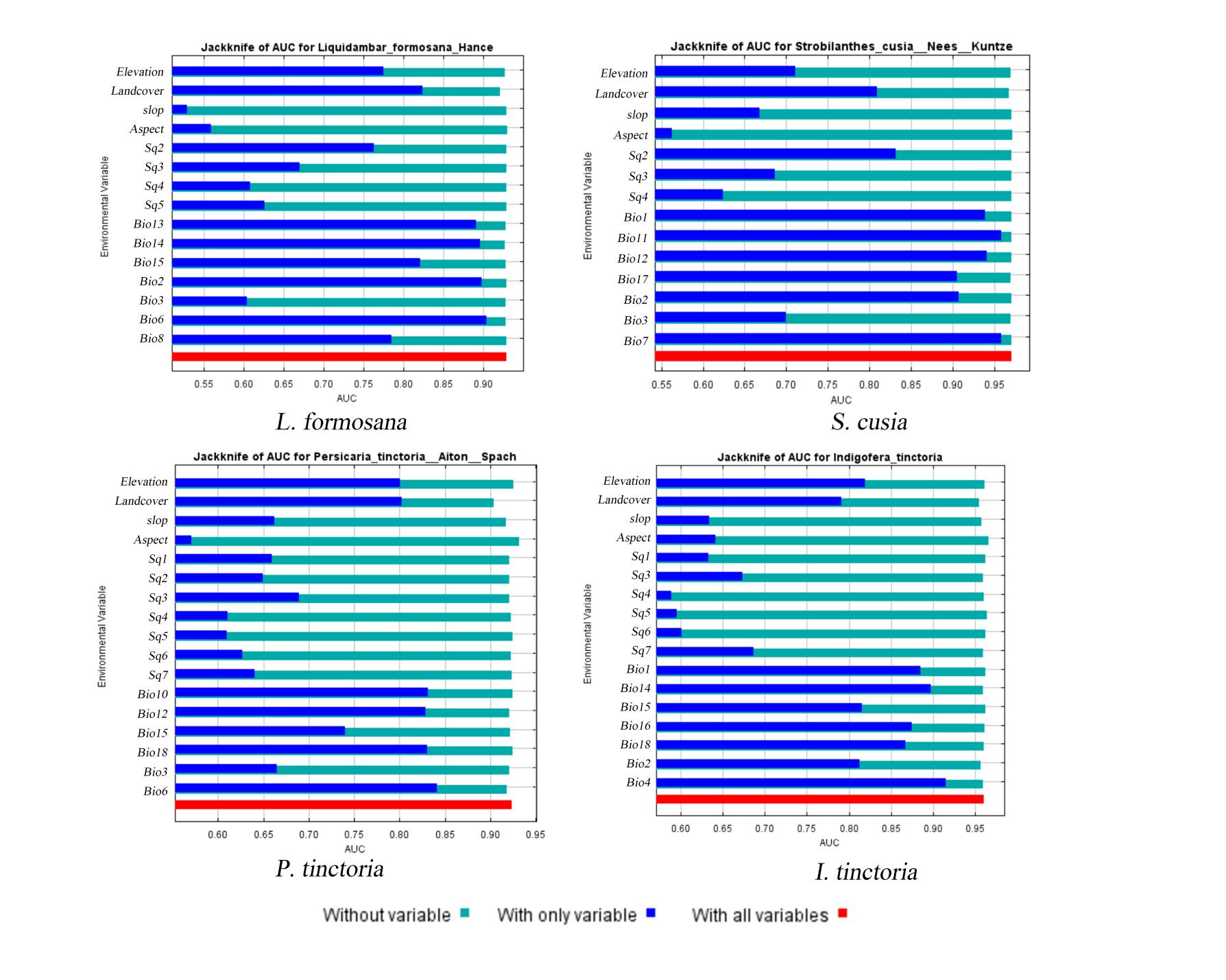


**Supplementary Figure 1.** Jackknife of AUC gain values of four plants.


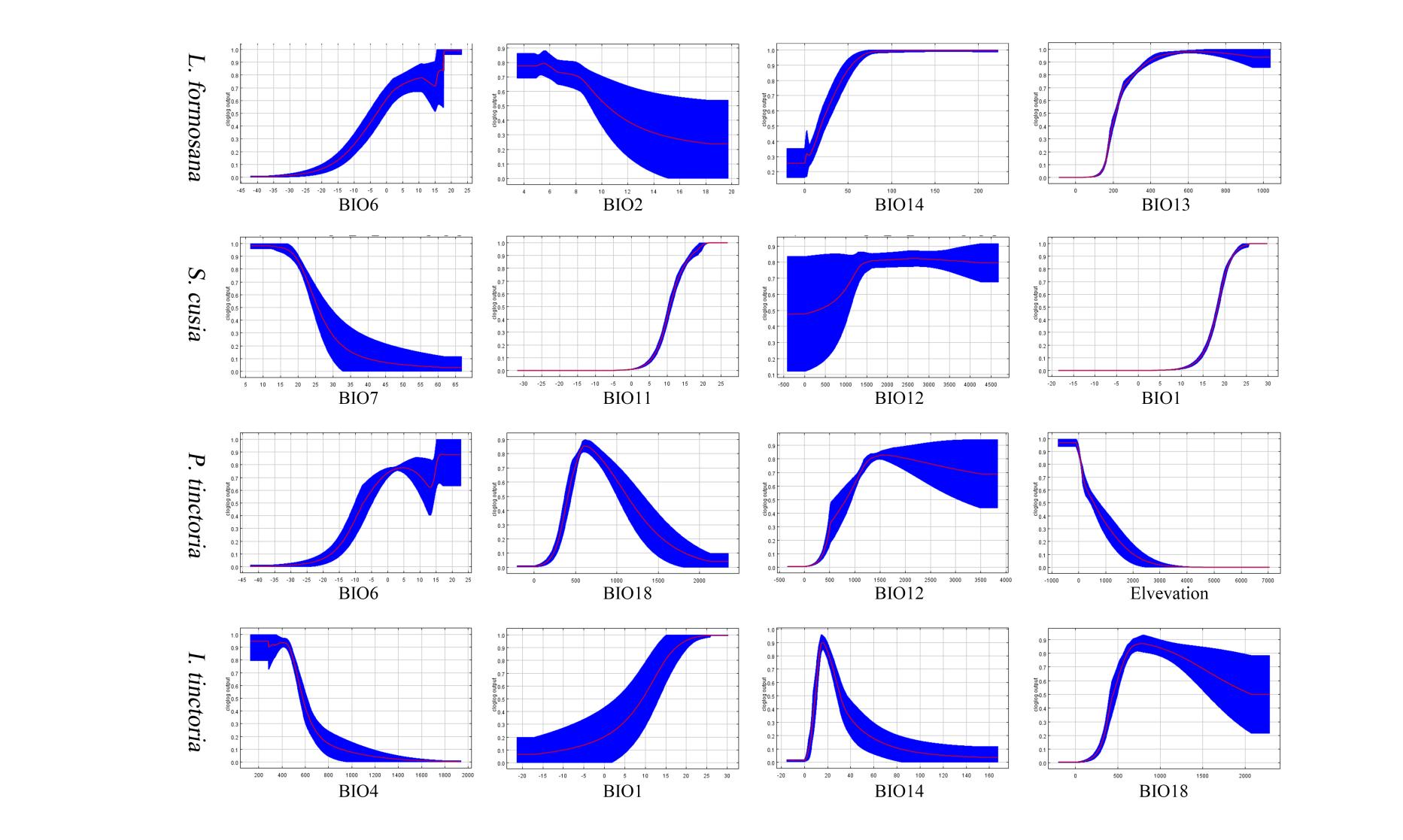


**Supplementary Figure 2.** Logistic output (Probability of pressence) of four plants.

## Supplementary Tables

**TABLE 1** Percent contribution and permutation importance of the environmental factors of *L. formosana*

| Variable | Percent contribution | Permutation importance |
| --- | --- | --- |
| Bio14 | 69.2 | 7.8 |
| Bio8 | 8.6 | 41.0 |
| Landcover | 7.8 | 6.1 |
| Bio2 | 3.2 | 12.4 |
| Elevation | 2.1 | 3.2 |
| Bio3 | 1.6 | 3.4 |
| Bio15 | 1.4 | 3.0 |
| Bio13 | 1.4 | 2.6 |
| Bio8 | 1.1 | 4.6 |
| slop | 0.9 | 0.8 |
| Sq2 | 0.6 | 2.1 |
| Sq3 | 0.6 | 3.0 |
| Aspect | 0.5 | 1.2 |
| Sq4 | 0.5 | 3.1 |
| Sq5 | 0.4 | 5.7 |

**TABLE 2** Percent contribution and permutation importance of the environmental factors of *S. cusia.*

| Variable | Percent contribution | Permutation importance |
| --- | --- | --- |
| Bio7 | 31.7 | 53.8 |
| Bio17 | 25.5 | 3.3 |
| Bio12 | 22.6 | 6.9 |
| Bio11 | 5.8 | 2.5 |
| Landcover | 4.9 | 3.6 |
| Bio1 | 3.00 | 4.8 |
| Bio3 | 2.3 | 4.2 |
| Slop | 1.7 | 0.9 |
| Elevation | 0.9 | 4.8 |
| Aspect | 0.5 | 0.3 |
| Bio2 | 0.4 | 3.9 |
| Sq2 | 0.4 | 3.2 |
| Sq3 | 0.2 | 0.9 |
| Sq4 | 0.1 | 6.8 |

**TABLE 3** Percent contribution and permutation importance of the environmental factors of *P. tinctoria.*

| Variable | Percent contribution | Permutation importance |
| --- | --- | --- |
| Iandcover | 17.4 | 6.7 |
| Bio12 | 16.1 | 2.2 |
| Bio6 | 16 | 13.4 |
| Slop | 12.1 | 5.7 |
| Bio18 | 12 | 1.5 |
| Elevation | 10.5 | 14.5 |
| Aspect | 3.0 | 1.0 |
| Sq3 | 2.3 | 2.7 |
| Bio15 | 1.9 | 0.4 |
| Bio3 | 1.9 | 1.3 |
| Sq5 | 1.5 | 7.6 |
| Sq1 | 1.3 | 2.3 |
| Sq4 | 1.1 | 3.8 |
| Sq2 | 0.9 | 12.1 |
| Bio10 | 0.7 | 4.6 |
| Sq7 | 0.7 | 1.3 |
| Sq6 | 0.6 | 18.9 |

**TABLE 4** Percent contribution and permutation importance of the environmental factors of *I. tinctoria.*

| Variable | Percent contribution | Permutation importance |
| --- | --- | --- |
| Bio4 | 33.2 | 19.3 |
| Landcover | 20.4 | 10.3 |
| Bio14 | 15.6 | 2.6 |
| Bio1 | 8.1 | 30.4 |
| Slop | 5.4 | 2.1 |
| Bio18 | 5.2 | 2.5 |
| Aspect | 2.6 | 2.8 |
| Bio2 | 2.1 | 2.8 |
| Bio15 | 1.3 | 0.7 |
| Sq3 | 1.3 | 1.2 |
| Sq6 | 1.1 | 7.6 |
| Sq4 | 0.9 | 1.8 |
| Sq7 | 0.7 | 2.3 |
| Sq1 | 0.6 | 3.1 |
| Elevation | 0.5 | 2.7 |
| Sq5 | 0.5 | 7.0 |
| Bio16 | 0.4 | 0.6 |
